# Reproductive concerns and its correlation with fear of recurrence and level of family support in patients of childbearing age with gynecologic malignancies

**DOI:** 10.1186/s12978-024-01827-9

**Published:** 2024-06-17

**Authors:** Xinying He, Ying Wu, Yaqing Zhou, Qin Chen, Xuping Li, Xuemei Fan, Chengjun Xia, Jiao Ma, Jing Han, Xue Han

**Affiliations:** 1 Women’s Hospital of Nanjing Medical University, Nanjing Women and Children’s Healthcare Hospital, Nanjing, 210004 Jiangsu China; 2 Hai’an Hospital of Traditional Chinese Medicine Affiliated to Nanjing University of Chinese Medicine, Nantong, 226600 Jiangsu China; 3 Department of Critical Care Medicine, Hai’an People’s Hospital, Nantong City, 226600 Jiangsu Province China; 4 General Hospital of Ningxia Medical University, Hui Autonomous Region, Xingqing District, Yinchuan, 750001 Ningxia China

**Keywords:** Childbearing age, Gynecological malignancies, Reproductive concerns, Fear of recurrence, Family support

## Abstract

**Background:**

To discuss the current status of reproductive concerns and its correlation with fear of recurrence and level of family support in patients of childbearing age with gynecologic malignancies.

**Methods:**

A convenient sampling method was used to enroll 188 patients with gynecologic malignancies in Nanjing Maternity and Child Health Care Hospital, Nanjing Drum Tower Hospital, General Hospital of Ningxia Medical University, and Haian Hospital of Traditional Chinese Medicine Affiliated to Nanjing University of Chinese Medicine from September 2022 to April 2023. Patients were assessed using general information questionnaire, Reproductive Concerns After Cancer Scale (RCAC), Fear of Cancer Recurrence Inventory (FCRI) questionnaire, and Perceived Social Support-Family (PSS-FA) Scale.

**Results:**

Among patients of childbearing age with gynecologic malignancies, the total RCAC score was (54.35 ± 7.52), indicating a moderate level of reproductive concerns. Patients scored (20.98 ± 4.51) on FCRI, implying a moderate level of fear of recurrence. The PSS-FA score was (9.57 ± 2.76), denoting a moderate level of family support. The total score and each dimensional score of RCAC were positively correlated with FCRI total score (*P* < 0.05), and negatively correlated with PSS-FA total score (*P* < 0.05). Fear of recurrence, family support level, number of children, educational background, treatment modality, and fertility intention were influencing factors for reproductive concerns in patients of childbearing age with gynecologic malignancies (all *P* < 0.05).

**Conclusion:**

The reproductive concerns, fear of recurrence and family support are all at moderate levels in patients of childbearing age with gynecologic malignancies, and reproductive concerns are positively correlated with fear of recurrence and negatively correlated with family support.

## Background

There were approximately 19.3 million new cancer cases and 2.3 million cancer deaths worldwide in 2020 [1]. The incidence rate of malignant tumors in women of childbearing age is 1–2% and is rising year by year, with a younger trend [2]. The continuous progress of malignant tumor prevention, diagnosis, and treatment technology, has greatly improved the 5-year survival rate of patients of childbearing age with gynecological malignant tumors. Gynecological cancers including cervical, ovarian, uterine and vaginal and vulvar cancer represent around 1 in 5 of all cancers diagnosed in women [3].The course of disease treatment may directly or indirectly affect the ovaries and uterus, leading to premature ovarian failure and amenorrhea, and other symptoms that adversely affect fertility [4]. With the opening of the "two child" and "three child" policies in china [5, 6], giving birth remains desirable among patients of childbearing age with gynecological malignancies who have not given birth at the time of diagnosis. Fertility anxiety refers to concerns about children's upbringing and fertility after an individual suffers from cancer. In some cases, it may pressure patients more than the disease itself, often lasting for several years, or even throughout the entire reproductive period [7, 8]. Fertility anxiety is a common problem among female patients with cancer, especially patients with gynecological malignant tumors. At present, research on the reproductive concerns of patients with gynecological malignant tumors is still in its infancy in China. This article investigates and analyzes the reproductive concerns, fear of recurrence, and family support levels of patients of childbearing age with gynecological malignant tumors, in order to identify important influencing factors of reproductive concerns. The aim is to attract the attention of domestic and foreign scholars and medical staff on the reproductive concerns of patients of childbearing age with gynecological malignant tumors and provide a reference for the development of scientific intervention measures in clinical practice.

## Materials and methods

### General information

The convenient sampling method was used to select 188 patients diagnosed with gynecological malignant tumors from September 2022 to April 2023 from Nanjing Maternal and Child Health Hospital, Drum Tower of Nanjing Hospital, Cardiovascular and Cerebrovascular Hospital of Ningxia Medical University General Hospital and Haian Hospital of Traditional Chinese Medicine affiliated to Nanjing University of Chinese Medicine. Inclusion criteria: (1) pathological diagnosis of cervical cancer, ovarian cancer, trophoblastic tumor, endometrial cancer, fallopian tube cancer, uterine sarcoma, and other gynecological malignancies; (2) age 18–45 years old; (3) clear awareness, able to communicate well, provided informed consent for study participation; (4) awareness of the condition and ensured stability. Exclusion criteria: (1) severe pulmonary, cardiovascular, and cerebrovascular complications in critical condition; (2) individuals with combined intellectual disabilities, mental illnesses, and language and hearing impairments; (3) expected survival time < 1 year; (4) primary infertility; (5) merge with other malignant tumors; (6) automatic withdrawal during the study period. Through the sample size estimation method [9]. The number of samples was calculated with power analysis [10, 11]. This study estimated 13 independent variables.The sample size is 10 times the number of independent variables and the sample was calculated in 0.15 effect sizes, 80% theoretical power, and 95% trust level.So the number of samples was determined as 188. All samples were reached. This study has been reviewed and approved by the Ethics Committee of the Maternity Hospital Affiliated to Nanjing Medical University (Nanjing Maternity and Child Health Hospital). The ethics acceptance number is 2022KY-135–02.

### Survey methods

#### Survey tools

1. General information questionnaire: The questionnaire was designed by the researcher for this study, including the patient's age, ethnicity, place of residence, marital status, number of children, educational level, per capita income of the family, occupation, medical payment method, tumor site and staging, treatment methods, fertility intention, etc.
2. Reproductive Concerns of Young Adult Female Cancer Scale (RCAC): The scale was developed by Gorman et al. [12] and translated into Chinese by Qiao et al. [13] It consists of 18 items, including 6 dimensions: pregnancy ability (3 items), pregnancy preparation (3 items), spouse knowledge (3 items), self-health (3 items), child health (3 items), and disease acceptance (3 items). Each item is scored on a scale of 1–5 points and 5 levels, with a total score of 18–90 points. The higher the score, the higher the level of fertility anxiety; 18–42 is low, 43–66 is medium, and 67–90 is high. The reliability and validity of the scale have been verified, and the overall Cronbach's α coefficient is 0.831.
3. The Fear of Cancer Recurrence Questionnaire (FCRQ): The scale was developed by Humphris et al. [14] and translated into Chinese by Zhang et al. [15], with a total of 7 items. The first 6 items are scored on a scale of 1–5 points and 5 levels, while the seventh item is scored on a scale of 1–10 points and 10 levels, with a total score of 7–40 points. A higher score indicates a higher level of fear of cancer recurrence, with 7–18 being low, 19–29 being medium level, and 30–40 being high. The reliability and validity of the scale have been verified, and the overall Cronbach's α coefficient is 0.892.
4. Perceived Social Support From Family (PSS-Fa): The scale was developed by Procidano et al. [16] and consists of 15 items. The answer to "yes" for each item is 1 point, "no" is 0 point, and the total score is 0–15 points. A higher score indicates a higher level of family support, with 0–5 indicating a low level, 6–10 indicating a medium level, and 11–15 indicating a high level. The scale has been validated for reliability and validity, and the overall Cronbach's α coefficient is 0.831.

### Data collection methods

All data were collected by online questionnaires. An investigation team was established, with all team members receiving unified and systematic training. Investigation was conducted after obtaining the consent of the hospital, department, and patients. The research purpose, significance, and filling methods were explained to patients and families. After the patient agreed to participate, the QR code was scanned to fill out the questionnaire. The patient was informed that the survey is conducted anonymously, and all questionnaires need to be filled out independently by the patient. If the survey was inconvenient for the patient to complete, the nurse would fill in for him after asking. The site and stage of the tumor was filled out by the nurse. If there were any questions during the filling process, the investigator provided unified answers and submitted the completed information. A total of 200 questionnaires were distributed, 12 invalid questionnaires were excluded, and 188 were recovered. The effective recovery rate of the questionnaire was 94%.

### Quality control

Relevant literature materials to the keywords "reproductive age", "gynecological malignant tumors", "reproductive anxiety", "fear of recurrence", "family support" were searched through channels such as "CNKI", "Wanfang", and "Pubmed". Experts from the educational institution were invited to randomly select 10 patients for pre-testing to prepare for the investigation and conduct tests to ensure the reliability, validity, and applicability of the scales used in this study. All questionnaires and the data sorting process of the scale were operated by two people, including data collection, inspection, verification, entry, etc., and 10% of the questionnaires were selected for recheck by a third person to ensure data integrity, consistency, and reduce the bias of results.

### Statistical methods

Data analysis was conducted using statistical software SPSS24.0, with counting data represented by “%”. Comparisons were performed by *χ*^2^-test, measurement data is presented as $\left(\overline{x }\pm s\right),$a t-test was performed, and one-way ANOVA was performed for multiple group comparisons. Pearson was used for correlation analysis, and multiple linear regression analysis was used for influencing factors. *P* < 0.05 indicates a statistically significant difference.

## Results

### Current status of reproductive anxiety, fear of recurrence and family support scores

The total score of reproductive anxiety (54.35 ± 7.52) in patients of childbearing age with gynecological malignancies was at a moderate level; recurrence fear (20.98 ± 4.51) was at a moderate level; and family support (9.57 ± 2.76) was at a moderate level, as shown in Table 1.

**Table 1:** Fertility worries, fear of recurrence, and family support scores of gynecological patients of childbearing age with malignant tumors (*n* = 188)

Project	Scoring range	Rating
Overall Fertility Worries Score	18–90 points	54.35 ± 7.52
Ability to conceive	3–15points	11.21 ± 2.62
Preparing for pregnancy	3–15 points	7.35 ± 2.25
Spouse Informed	3–15 points	7.67 ± 2.14
Own health	3–15 points	9.87 ± 2.32
Child health	3–15 points	9.52 ± 2.55
Disease acceptance	3–15 points	7.96 ± 2.43
Total Recurrence fear score	7–40 points	20.98 ± 4.51
Total Family support score	0–15 points	9.57 ± 2.76

### Correlation between reproductive anxiety, fear of recurrence and family support

The Pearson correlation analysis results showed that the total score of reproductive anxiety and various dimension scores were positively correlated with the total score of relapse fear (*P* < 0.05), and negatively correlated with the total score of family support (*P* < 0.05), as shown in Table 2.

**Table 2:** Correlation between fertility worry, recurrence fear and family support in patients of childbearing age with gynecological malignant tumors

Indicators	Relevance	Overall fertility Worries score	Pregnancy Ability	Preparing for pregnancy	Spouse Informed	Self- Health	Children health	Illness Acceptance
Total Recurrence Fear Score	*r*	0.392	0.286	0.344	0.318	0.276	0.251	0.309
	*P*	< 0.001	< 0.001	< 0.001	< 0.001	< 0.001	< 0.001	< 0.001
Total Family support score	*r*	-0.338	-0.282	-0.290	-0.271	-0.307	-0.311	-0.288
	*P*	< 0.001	< 0.001	< 0.001	< 0.001	< 0.001	< 0.001	< 0.001

### Single factor analysis of reproductive anxiety

The results of univariate analysis showed that there was a statistically significant difference in the scores of reproductive anxiety among patients of childbearing age with gynecological malignancies who had different numbers of children, educational level, treatment methods, and fertility intentions (*P* < 0.05). Different age, ethnicity, place of residence, marital status, per capita family income, occupation, and medical payment methods showed no statistically significant differences (*P* > 0.05) in reproductive anxiety scores among patients of childbearing age with gynecological malignant tumor in terms of tumor site and staging, as shown in Table 3.

**Table 3:** Univariate analysis of fertility worries in patients with gynecologic malignant tumors of childbearing age (*n* = 188)

Item	n	Fertility worries score ($\overline{x} \pm s$, points)	*t*/*F*	*P*
Age (years)			0.111	0.912
< 35	52	54.03 ± 6.72		
≥ 35	136	53.91 ± 6.63		
Ethnicity			0.543	0.588
Han	180	54.36 ± 6.51		
Other	8	53.88 ± 6.74		
Place of Residence			1.253	0.212
City	145	53.23 ± 6.52		
Rural	43	54.68 ± 7.14		
Marital status			0.650	0.523
Unmarried	14	52.32 ± 5.22		
Married	152	54.21 ± 6.05		
Divorced	22	53.96 ± 5.58		
Number of children			4.91	0.008
0	31	56.96 ± 7.18		
1	124	54.33 ± 6.83		
≥ 2	33	51.62 ± 6.39		
Educational level			5.57	0.0045
Elementary/Primary education	12	56.78 ± 6.35		
High school	25	54.12 ± 7.32		
College and above	151	51.32 ± 6.24		
Per capita household income(CNY)			0.42	0.738
≤ 3000	15	55.22 ± 6.34		
3000–5000	44	56.01 ± 7.21		
5000–10000	69	54.58 ± 7.43		
≥ 10,000	60	54.72 ± 6.55		
Occupations			0.950	0.343
In-service	152	53.25 ± 5.88		
Quit/none/retired	36	54.30 ± 6.32		
Medical payment method			0.08	0.923
Medicare	132	54.24 ± 6.82		
New Rural Cooperative Medical	38	53.76 ± 6.43		
Own expense	18	54.16 ± 6.22		
Tumor site			0.93	0.425
Cervical cancer	84	54.89 ± 6.45		
Endometrial cancer	48	53.23 ± 6.01		
Ovarian cancer	22	54.48 ± 6.36		
Other	34	55.41 ± 7.32		
Clinical staging			0.673	0.422
I + II	123	54.27 ± 6.21		
III + IV	65	53.88 ± 5.66		
Treatment style			6.04	0.0029
Surgery	94	56.98 ± 7.23		
Chemoradiotherapy	73	55.51 ± 6.61		
Other	21	51.22 ± 6.46		
Fertility willingness			3.811	0.0002
Yes	145	56.45 ± 7.82		
None	43	51.49 ± 6.25		

### Multiple regression analysis of reproductive anxiety

The level of reproductive anxiety in patients of childbearing age with gynecological malignant tumors was used as the dependent variable. The variables of recurrence fear and family support in correlation analysis and meaningful children's number, education level, treatment method, and fertility intention in univariate analysis were used as independent variables. The variable assignments are shown in Table 4. The results of multiple linear regression analysis showed that fear of recurrence, family support, number of children, educational level, treatment methods, and fertility intention were all influencing factors for fertility anxiety in patients of childbearing age with gynecological malignancies (*P* < 0.05), as shown in Table 5.

**Table 4:** Assignment of independent variables in multiple linear regression analysis

Independent variable	Type of variable	Assignment situation
Relapse fear	Continuous type variables	The original value is substituted
Family Support	Continuous type variables	The original value is substituted
Number of children	3 Categorical variables	≥ 2 = 1 (dummy variables); 1 = 2; 0 = 3
Education level	3 Categorical variables	Junior college and above = 1(dummy variable); High school = 2; Elementary/middle school = 3
Type of treatment	3 Categorical variables	Other = 1 (dummy variables); Chemoradiotherapy = 2; Surgery = 3
Fertility desire	2 Categorical variables	None = 1; Yes = 2

**Table 5:** Results of multiple regression analysis of fertility worries in patients of childbearing age with gynecologic malignancies

Variables	Partial regression coefficient *B*	SE	Standard coefficient	*T*	*P*	95% confidence interval
Constant	-3.923	0.463		-8.487	< 0.001	-4.801–2.911
Relapse fear	0.026	0.004	0.457	7.042	< 0.001	0.015–0.038
Family Support	-0.021	0.005	-0.273	-3.266	0.001	-0.040–0.007
Number of children	0.015	0.004	0.282	3.657	< 0.001	0.003–0.022
Education level	0.040	0.009	0.316	4.108	< 0.001	0.017–0.058
Treatment mode	0.019	0.005	0.199	3.514	0.001	0.010–0.034
Fertility intention	0.029	0.008	0.429	4.039	< 0.001	0.009–0.044

## Discussion

### Analysis of the current status of reproductive anxiety, fear of recurrence and family support

The results of this study show that the total score of fertility anxiety (54.35 ± 7.52) of patients of childbearing age with gynecological malignancies is at a medium level, which is higher than that of patients at childbearing age with breast cancer (49.05 ± 15.64) in the study by Hailing et al. [17], and lower than that of patients with cervical cancer in the study by Wang et al. [18], indicating that differences in fertility anxiety level may be dependent on the subjects selected. Most studies show that [19, 20], fertility concerns of patients with gynecological malignancies such as cervical cancer and ovarian cancer are at a high level, mainly due to possible hysterectomy and ovary removal during treatment, which will directly affect fertility. At the same time, influenced by the traditional concept of "inheriting the family line" in China, it is believed that women bear the responsibility of pregnancy, childbirth, and other responsibilities. Thus, women are more prone to reproductive concerns after suffering from gynecological malignancies and have high levels of reproductive concerns [21]. However, most of the patients in this study have children, with 31 cases having no children, accounting for only 16.49%. Under the current high cost of childbirth and social pressure, those with children have lower willingness to give birth compared to those without children, resulting in a relatively low overall anxiety in the middle to lower levels. Additionally, we found that among all dimensions of reproductive anxiety, the highest score was for pregnancy ability (11.21 ± 2.62), which may be due to the fact that childbirth is an important component of building a harmonious family and maintaining marital relationships. Therefore, patients of childbearing age with gynecological malignancies are most concerned about their own pregnancy ability. Medical staff should pay attention to the psychological care of patients of childbearing age with gynecological malignant tumors, and provide effective measures to alleviate their reproductive anxiety.

The results of this study show that the total fear of recurrence score (20.98 ± 4.51) of patients of childbearing age with gynecological malignancies is at a moderate level, which is similar to the findings of Zhang et al. [22]. Cancer recurrence fear refers to the psychological fear of patients with cancer towards the recurrence, metastasis, and progression of cancer in other or primary areas. It is mainly manifested as excessive tension, examination, and attention to changes in their own symptoms, and understanding symptoms such as chest tightness and pain as signs of worsening the condition [23]. Female patients are generally more perceptive, and after learning of their own illness, their psychological burden increases, and they are more concerned about disease recurrence after treatment. At the same time, some gynecological malignancies have a higher degree of malignancy, making patients' fear of cancer recurrence more significant. Therefore, medical staff should provide patients with psychological counseling, explain disease related knowledge, and reduce their fear of cancer recurrence.

The results of this study show that the total family support score (9.57 ± 2.76) of patients of childbearing age with gynecological malignancies is at a moderate level. At present, there is no comparison of family support data for patients of childbearing age with gynecological malignant tumors in China. In the study of elderly patients with hypertension by Li et al. [24], the family support score was at a moderate level (9.51 ± 1.68). Family support is an important component of social support, which is closely related to the mental health of patients [25]. This study shows that family support for patients of childbearing age with gynecological malignancies is at moderate to upper levels, indicating a high level of support provided by the patient's family. This helps to improve the patient's psychological adaptability and promote mental health. Therefore, medical staff should inform and encourage patients' families, provide them with spiritual and material support, and improve their family support scores.

### Analysis of the correlation between reproductive anxiety, fear of recurrence and family support

The results of this study show that the total score of reproductive anxiety and its various dimensions are positively correlated with the total score of recurrence fear, and negatively correlated with the total score of family support. Recurrence fear and family support are both influencing factors for reproductive anxiety in patients of childbearing age with gynecological malignancies, which is consistent with the results of Zhang et al. [26] and Wang et al. [27]. Cancer, chemotherapy, surgery, and other related factors are inherently traumatic stress for patients, which can lead to negative psychological emotions such as anxiety and irritability, affect treatment compliance, and increase patients' concerns and fears about subsequent treatment [28]. Among patients of childbearing age with gynecological malignancies, the higher the level of fear of cancer recurrence, the more worried they are that the disease will further affect their own health, pregnancy ability, and other reproductive functions, thereby increasing the level of reproductive anxiety. Family support plays an important role for patients, providing them with strong spiritual support, helping establish confidence in overcoming the disease, alleviating negative emotions, and ultimately reducing the level of reproductive anxiety. Therefore, medical staff should strengthen the assessment of reproductive concerns, fear of recurrence, and family support for patients of childbearing age with gynecological tumors, and develop scientific intervention plans to effectively improve the level of family support and reduce the level of reproductive concerns and fear of recurrence. At present, there are few research reports on the correlation between reproductive anxiety, fear of recurrence, and family support in China. The results of this study require further analysis and verification in the future.

### Analysis of influencing factors of reproductive anxiety

In addition to the fear of recurrence and family support, the number of children, educational level, treatment methods, and willingness to have children are all influencing factors for reproductive anxiety in patients of childbearing age with gynecological malignant tumors. This is consistent with the findings of Chen et al. [29] and Tan et al. [30]. According to the analysis of the number of children and fertility desire, female patients who have not given birth and those who have fertility desire have a higher level of fertility anxiety, influenced by the traditional concept of "carrying on the family line" in China. Women who have not given birth and those who have fertility desire are under greater pressure to give birth. In addition, the policy of encouraging "two children" and "three children" can significantly increase the level of fertility anxiety of patients. At the same time, patients who already have a child and still have a reproductive need may have concerns about whether they can continue to have children after suffering from cancer. From the perspective of educational level, those with low educational level have limited access to disease-related knowledge and insufficient understanding, which leads to incorrect understanding of disease treatment, recurrence, and fertility, causing difficulty in reproductive decision-making and raising the level of reproductive anxiety. From the perspective of treatment methods, patients with gynecological malignant tumors often undergo surgery, chemotherapy, radiotherapy, and other methods of treatment. Surgery impacts the sexual organs, causing patients to experience anxiety about fertility; chemotherapy can lead to premature ovarian failure and even temporary or permanent infertility; radiotherapy can cause pathological changes such as endometrial damage and myometrium fibrosis, affect the ovary, lead to sexual dysfunction, and therefore affect the level of fertility anxiety. Medical staff should focus on the influencing factors, try to completely understand the patient's family situation, cultural background, reproductive needs, and other content to take effective measures to reduce the level of reproductive anxiety, such as promoting and educating on various forms of diseases and fertility, providing effective psychological care, and carrying out reproductive protection and egg preservation.

## Conclusions

In summary, patients of childbearing age with gynecological malignant tumors have moderate levels of fertility concerns, fear of recurrence, and family support. Fertility concerns are positively correlated with fear of recurrence, and negatively correlated with family support. There are many influencing factors for fertility concerns, and more attention should be paid to patients who have no children, low education, surgery, chemotherapy, and desire to have children. We recommend that scientific interventions be developed for gynecological malignancies patients of childbearing age to reduce levels of fertility concerns. This study only selected four tertiary hospitals for investigation and research. The sample size and representativeness of the samples is limited, and there may be regional differences. In the future, the sample size can be expanded to further explore the factors related to reproductive anxiety in patients of childbearing age with gynecological malignancies.

## Data Availability

The datasets generated during and/or analyzed during the current study are available from the corresponding author on reasonable request.
